# 

**Published:** 1811-12

**Authors:** 


### MONTHLY CATALOGUE OF MEDICAL BOOKS.

A Treatise on the Diseases of Children, with Directions for the Management of Infants from the Birth. By Michael Underwood, M. D. Licentiate in Midwifery of the Royal College of Physicians in London, Physician to her Royal Highness the Princess of Wales, and late Senior Physician to the British Lying-in Hospital. In 3 Vols. The Sixth Edition, Revised and Corrected

Practical Observations on various novel modes of operating on Cataract, and of forming an artificial pupil. By Robert Muler Holbeach. 8vo.

Observations on the Cataract and Gutta Serena; including a Translation of Wenzel's Treatise on the Cataract; a new Chapter on the Operation of largely puncturing the Capsulæ of the Crystalline Humour; and many additional Remarks on the Gutta Serena. By James Ware, F. R. S.

Observations on the Use of Caustic Alkali in Scrofula, and other Chronic Diseases. By Joseph Brandish. 8vo.

Practical Observations on the Treatment of the Diseases of the Prostate Gland. Illustrated by Copper Plates. By Everard Home, Esq. F. R. S. 8vo.

---

### NOTICES TO CORRESPONDENTS.

Mr. Knowles, in reply to a Norfolk Practitioner; Mr Spencer on Cynanche Trachealis; Studiosus Medicinæ, &c. &c. in our next.

We are grateful to Mr. Machell for his excellent cases in support of bleeding in Scarlatina Anginosa; to which we earnestly request our readers attention; and that, as the practice is hardly yet sufficiently established, they will favour us with the resnlt of their experience on this most important subject.

We also solicit Communications from Gentlemen residing in our Colonies, or whose military or naval duties may lead them to distant quarters of the globe, on the progress of Vaccination; for the question is yet augmenting in interest and importance.

The letter of Mr. Pearson, in our present Number, is highly satisfactory.

From Mr. Mann's Account of New South Wales, published this year, we learn that Vaccination was successfully introduced with the colony there, in 1804; but that it has since been wholly lost. On this subject we entreat information. It appears that Small-pox at intervals produces the most dreadful ravages amongst the natives of New South Wales, and is held in so much horror by them, that they immediately desert, and suffer to perish without assistance whoever is unfortunate enough to be seized with the malady. Perhaps this may be the most effectual means of preventing the infection from spreading, but humanity revolts from the practice; and it at least demands the attention of Government to cause the effectual re-introduction of Vaccination amongst those unhappy people.

### INDEX.

ACACIA, peculiar species of 477
Acid, on the preparation of carbonic 95
Acids, prize question on the decomposition of 168
Aconite, experiments with the leaves of 133, 139
Action of the vital power, on the 1
——— of vessels in inflammation, on the 353
Actions interrupted by the voluntary power 98
———, of stimulated 100
Aether, method of dissolving elastic gum in 382
Affection, on a case of nervous 458
Agile lizard, description of the 27
Air bubbles in the blood vessels of dead persons 405
Alcohol, experiments concerning 129
——— of wine, on the 168
Alkalies, on the decomposition of acids, &c. ib.
Alkalies, on the metals of fixed 18
Allan, Mr. on the rocks near Edinburgh ib.
Almonds, on the essential oil of bitter 132, 138
Amenorrhœa, efficacy of rubia tinctorum in 107
America, South, interesting account of 10
American philosophical register, account of 250
——— medical topography, statement of 421
Anatomy, present state of 6
Anatomy, on surgical 388
Aneurism, on the operation for popliteal 241
——— of the aorto, case of 251
Animals, analogy between plants, &c. 8
Animal food, reasons for not eating 310
Aorta of dogs, experiments on the 83
Aorta, case of aneurism of the 251
Apoplexy, case of 181
Apothecaries, on the practices of 385, 455
Appoo, Don Juan, a Cingalese physician, anecdote of 491
Aqueous injection of hydrocele, on 110
Arsenic, in tic douloureaux, on 494
Arsenic, observations on 31
Arundo, on the 477
Ascites, on a case of 388
Asclepias asthmatica, description of the 30
Asthma, virtues of datura in 150, 491
———, efficacy of bleeding in 154
Augite, on the situation of the 305
Augsburg, peculiarity in brewing at 546
Baillie's, Mr. case of suppression of urine 161
Balfour's, Mr. case of rupture ib.
Bark, on the various kinds of 325
Barton, Dr. on the natural history of the opossum 422
Bate's tincture, account of 191
Batemen, Dr. on the progress of vaccination 390
Beaver's, Dr. case of cynanche trachealis 377
Beddoes, Dr. on the opinions of 391
Beer, the herb bennet used in 342
Belladonna, experiments on 252
——— in hooping cough, efficacy of 510
Bennet, the herb used in beer 342
———, Mr. account of his garden 476
Biography, enquiry concerning a medical 456
Blenheim-street medical society, account of the 504
Blood, on its circulation in parts inflamed 354
——— of diabetic patients, on the 463
Blood letting in fever, on 108
——— in asthma, success of 154
——— in diabetes, on 437
Blood vessels, effects of gases injected into 252
———, on the dilatation of the 435
———, air bubbles in the 405
Body, experiments made in the cavity of the 167
BOOKS, NEW MEDICAL, CRITICAL ANALYSIS OF.—A Letter on the Study of Medicine and on the Medical Character; by Peter Reid, M. D. 68. Essay on the Operation of Cutting for the Stone. By C. B. Trye, 72. The Edinburgh Medical and Surgical Journal, 151. Hortus Elginensis; or, a Catalogue of Plants cultivated in the Elgin Botanic Garden, New York, by David Hosack, M. D. 162. Medical Papers communicated to the Massachusett's Medical Society, 214. A Letter to Dr. jones on the Composition of the Eau de Medicinale, by James Moore, 224. Practical Observations on the Diseases of the Human Eye, by Joseph Reade, M. D. 227. Report of the National Vaccine Establishment, 234. A Letter to the Commissioners for Transports, &c. on the Operation for Popliteal Aneurism; by A. C. Hutchison, M. D. 241. A posological Companion to the London Pharmacopœia, by John Nott, M. D. 246. De la Methode Jatraleptique; et sur Nouveau Remede dans le traitement des Maladies veneriennes & Lymphatiques; par J. A. Christien, M. D. 314. The London Dispensatory; by A. T. Thompson, 321, 394. The Edinburgh Medical and Surgical Journal, No. xxvii, 337. A Treatise on Surgical Anatomy; by Abraham Colles, 388. Journal generale de Medicine, &c. redige par M. Sedillot, &c. 402, 482. Recherches de Physiologie et de Chimie pathologiques; par P. H. Nysten, M. D. 405. Of the Medicinal Leech, by M. Vitet, 408. A Treatise on Gout; by John Ring, 478. Report of the Broad-street Vaccine Institution, 488. The Edinburgh Medical and Surgical Journal, No. xxxvii. 490. Practical Observations on Cancer, by John Howard, 501.
Books, monthly catalogue of new medical 84, 175, 264, 352, 424, 518
Bostock, Dr. on the cactus coccinillifer 342
Boston in New England, history of medicine at 215
———, medical school established at 217
Botanical reports 170, 256, 425, 512
——— lottery announced 345
Botany, on the study of 291
———, historical notes relative to 470
Brain, its influence on the heart 49
———, influence of poisons on the 142
Brande, Mr. on the alcohol of wine 168
Brodie, Mr. on the influence of the brain 49
———, on vegetable poisons 129
———, observations on the experiments of 278
Brussels, prize questions of the society of 345
Bullock's, Mr. museum, account of 16
Burn, case of deformity by a 158
Burns, facts and observations on 499
Burroughs, Mr. on the eau medicinale ib.
Cactus coccinillifer, analysis of the 342
Calomel in rheumatism, on 210
Caloric, on the excess of 1
Camelopardalis giraffe, description of the 17
Camphor, its effects in suppression of urine 161
Cancer, efficacy of the lizard in 25
———, practical observations on 501
Canton, state of vaccination at 453
Caoutchouc, method of dissolving 264, 381, 451
Carbonic acid, on the preparation of 95
Cases—Of polypus vaginæ, 38; exomphalocele, 40; colica pictonum, 43; asthma, 49, 55, 56; Iscuria vesicalis, 75; small-pox after vaccination, 82, 177, 234, 397; small-pox, 83, 236, 249, 379, 397, 423; suppressed catamenia, 106. On fever, 115, 282; paralysis of the lower extremities, 153; hæmorrhœa petechialis, ib. Of bleeding in cough, 154; of the larvæ of an insect voided in urine, 154; poisoning by foxglove, 155; poisoning by corrosive sublimate, 156; enlargement of the heart, 157; deformity of the face by burning, 158; of rupture of the lungs in parturition, 161; suppression of urine, ib.; suffocation, 162; apoplexy, 111; diarrhœa, 183; gout, 194, 244, 355, 369, 499; tinea capitis, 203; hydrophobia, 206; rheumatism, 210; ophthalmia, 230; popliteal aneurism, 242; fever, 250; aneurism of the aorta, 251; puerperal fever, 265; acute rheumatism, 277; schirrhous tumor, 320; raptured spleen and liver, 337; erythema mercurialis, 341; inflammation, 360; ulcer, 364, 5; fractured spine, 371; ascites, 388; tetanus, 402; hæmorrhage, 122; typhus, 433; scarlatina anginosa, 443; spontaneous ptyalism, 452; annotations of various, 455; nervous affection, 458; somnambulism, 483; asthma, 490; tic douloureux, 494; tumor of the tongue, ib.; diabetes, 467; cancer, 503; hydatids, 506.
Catamenia, case of suppression of the 105
Cathartics in the gout, on 380
Catheters, use of large 205
Cavity of the body, on wounds in the 167
Ceylon, vaccination at 393
Chemistry, on the progress of 18
———, on the advantages of 389
Chester's, Dr. account of a fœtus 167
Chisholm's, Dr. cases of ruptured spleen and liver 337
Chlorosis, efficacy of rubia tinctorum in 105

Christien's, Dr. facts and experiments 315
Christie, Dr. on the effects of datura 161
——— on diabetes melitus 491
Cinchona, description of 12
———, varieties of the 325
Clarke's, Dr. medical report 151
Climate, on the influence of 14
Clough, Dr. case of exomphalocele 40
Cold applications, on 433
Colica pictonum, case of 43
Colles, Mr. on surgical anatomy 388
Collinson, Mr. biographical notices of 470
Color, influence of the climate on the 14
Comet, account of the 352, 429
Condor, description of the 15
Correspondents, notices to 88, 170, 264, 352, 430, 518
Corrosive sublimate, case of poison by 156
Croupe, successful remedy for the 509
Cynanche trachealis, case of 377, 455
Datura, on the virtues of 21, 160
Davidson's, Mr. forceps described 510
Davies, Mr. on rheumatism 277
———, on spontaneous ptyalism 452
Davy's, Mr. discoveries account of 18
———, experiments on oximuriatic gas and oxigen 298
Death from vegetable poisons, on 129
———, observations and enquires on 405
Deformity of the face, case of 159
Diabetes, on blood in the sugar of 462
———, success of bleeding in 497
———, melitus, notes on 491
Diarrhœa, case of 183
———, on the treatment of 511
Dickson's, Dr. case of apoplexy 181
Diseases in London, reports of 84, 256, 348, 511
Diuretic liniment, account of 422
Dogs, on the aorta of 83
Eau midicinale, on the 23, 185, 193, 385, 499
———, Mr. Moore on the composition of 224
Edinburgh Medical Journal, analysis of 151, 337, 490
———, proceeding of royal society of 168
———, prize questions of the medical society at 169
———, Harveian society at ib.
Eels, on the migration of 516
Egg, on the phisiology of the 7
Elastic gum, on dissolving the 382, 451
Electricity, on medical 109
Elgin garden, New York, description of the 162
English, Mr. on asthma 499
Enquiry concerning Dr. Milward 456
Epiphora, observations on 232
Epsom salts, method of preparing 251
Erysipelas, on cold applications in 433
Erythema mercuriale, case of 341
Exomphalocele, case of 40
Experiments, on the brain and heart, 59; on the aorta of dogs, 83; on sensation, 89; on vegetable posons, 129; on wounds in the cavity of the body, 167; on the combination of gases, 298; on sodalite, 303; on the circulation of the blood, 354, on the blood vessels, 406; on worms, 454; on the blood in diabetes 463
———, observations on Mr. Brodie's 278
Extremities, apparent paralysis of the lower 153
Eye, on the focal distance of the 247
———, on diseases of the 227
Face, case of deformity of the 159
Female, description of an African 16
Fever, on typhus 84, 152
———, on blood letting in 37, 108
———, on spotted 217
———, on puerperal 265
Fire, case of persons perishing by the gas of coal 162
Fistula lachrymalis, on the treatment of 228
Fœtus, account of a remarkable 167
Fogo, Mr. on twin cases 115
———, reply to 282
Food, reasons for abstaining from animal 310
Forceps, on the use of the 111
———, description of a new 510
Fordham, Mr. case of a polypus vagina 38
Fothergill, Dr. anecdote of 456
Fox-glove, case of poison by 155
Fractured thigh, on the treatment of 120
Fractured spine, case of 371
Gases injectured into the blood 252
Generation, on equivocal 454
Gibbes, Dr. on stramonium 49
Giraffe, account of the 17
Gold, medicinal uses of 321
Gout, on the eau medicinale in 23, 499
———, on cold applications in 96
———, on the treatment of 188
———, case of 244
———, on cathartics in 380
———, on the cure of 481
———, rhododendron recommended in 508
Grenville, Dr. on rubia tinctorum 105
Gryphon, on the existence of the 16
Guarinis, remarkable account of the 13
Gum, on dissolving the elastic 382, 455
Hair, variation in the state of the 308
Hamilton's, Mr. theory of worms 273
Harlem royal society, proceedings of the 76, 169

Harrison's, Dr. bill, account of 2
——, observations on 123
Harold, Mr. on fractured thigh 120
——, on fractured spine 371
Harrup's, Mr. case of colica pictonum 43
——, on tinea capitis 203
——, on acute rheumatism 449
Harveian society, questions of the 169
Hawthorn, vulgar notion concerning the 350
Heart, influence of the brain on the 49
——, on an enlargement of the 157
Heat, on the generation of animal 58
Henderson, Dr. on medical biography 456
Henry's, Dr. experiments 19
Hepatic diseases the cause of hypochondriasis 212
Hepatitis, case of 153
Herpes, on the prepuce, observations on 495
Hill's, Mr. case of burn 158
Historical sketch of the progress of medicine 1
Hæmorrhœa petechialis, case of 153
Hæmorrhage, cases of 422
Hooping cough, treatment of 85
——, belladonna recommended in 510
Horse, snake found in the eye of a 273
Hosack's, Dr. botanic garden, account of 162
——, philosophical register noticed 251
Houzoanas, remarkable peculiarity in the 16
Howard's, Mr. observations on cancer 501
Humboldt's travels, account of 12
Hume, Mr. on eau medicinale 185
Hutchinson, Dr. on popliteal aneurism 240
Hydatids, case of 506
Hydrocele, on aqueous injection in 110
Hydrophobia, on 206
Hypochondriasis, cause of 212
Indians, South American, on the 14
Inflammation, on the action of the vessels in 353
—— of the neck of the bladder, case of 161
Injections, on aqueous in hydiocele 110
——, improper use of 161
Inoculation, on small pox 117, 207
——, cases of small pox after 238
Insect, the larvæ of, found in urine 156
Instrument, description of a tooth 167
Introsusception, case of 250
Ischuria vesicalis, case of 75
Jackson, Dr. on the practice of 37
Jones, Dr. letter to 224
Journal Generale de Medicine, contents of 402, 482
Kiesser's, Dr. diuretic liniment 422
King's, Mr. cases of suffocation 162
Knight's, Mr. experiments on trees 8
——, theory of vegetation 170
Knowles's, Mr. case of diarrhœa 183
——, remarks on 387
Lacerta, on the genus 25
——, agilis, a remedy for cutaneous complaints 250
Lamprey, account of the 262
Larvæ of an insect found in urine 154
Laudanum, case of recovery from an excessive dose of 495
Lectures announced—Mr. Brookes on anatomy, phisiology, and surgery, 252; at the theatre of anatomy, Great Windmill-street, 253; St. Thomas's and Guy's Hospitals, ib.; Mr. Stevenson on diseases of the eye and ear, 254; Mr. Shute, on anatomy, phisiology, and surgery, ib.; Dr. Buxton on the practice of medicine ib.; Dr. Clutterbuck on the theory and practice of the physic, ib.; Dr. Reid on the theory and practice of Medicine, ib. Dr. Tuthill on the practice of physic and chemistry, ib.; Dr. Adams on the institutes and practice of Medicine, ib.; at St. George's Hospital, by Dr. Pearson, 255; Dr. Clough on midwifery, ib.; Dr. Clarke and Mr. Clarke on ditto, ib.; Mr. Moor on the teeth, ib.; Dr. Ramsbotham on Midwifery, ib.; Dr. Thynne on midwifery, ib.; Dr. Dennison on ditto, ib.; Mr. Taunton on anatomy and surgery, 346; Mr. Carlisle on surgery and phisiology, ib.; Dr. Watts on the practice of medicine, ib.; Mr.—on anatomy and surgery, 347, Dr. Paris on pharmaceutical chemistry, ib.; Dr. Merriman on midwifery, ib.; Dr. Squire on ditto, ib. At the Scientific Institution, Prince's-street, Cavendish-square, 423; Mr. Pearson on syphilis, &c. ib.; Mr. Singer on electricity, 511; Dr. Squire on midwifery, ib.
Leech, on the medicinal 408
Lemon trees first introduced into England 475
Lineament, description of a diuretic 422
Lithotomy, observations on 285
Liver, on the word 312
——, of a ruptured spleen and 337
Loffling, Peter, account of 9
London, reports of diseases in 84, 256, 348, 511
——, deaths from small pox in 509
Lottery, account of the botanical 245
Lues venerea, observations on 424

Lungs, case of rupture of the 161
Lyall, Mr. on burns 499
Lycæum medicum Londinense, on the state of the 384
Lymington, method of making sulphat of magnesia at 251
Machell's, Mr. cases of scarlatina anginosa 442
M'Cullock, Mr. on bleeding in fever 108
Madhouses, on the act for regulating 148
Magnesia, method of making sulphat of 251
M'Keur's, Mr. case of diabetes 497
Man midwife, on the origin of the term of 41
Mania, cases of 317
Marcet, Dr. on sugar in the blood 467
Massachusett's Medical society, account of the 214
Medical and philosophical intelligence 76, 167, 247, 344, 417, 504
——— electricity, on 109
——— school at Boston 247
——— publication, catalogue of 84, 175, 264, 352, 424, 518
——— Benevolent Society, account of 457, 510
Medicine, historical sketch of the progress of 1
———, on the study of 68
Medicines, on the practice of sending in 385, 455
Mellor, Mr. on apparent paralysis 153
Melon, on the culture of the 170
Mercury, remarks on the uses of 27
——— in acute rheumatism, on 277
———, tumours of the tongue cured by 494
Meteorological reports 17, 175, 263, 351, 429, 517
Mexico, statistical account of 11
Miller, Dr. on the history of physic 5
Milward, Dr. enquiry concerning 456
Mineral, analysis of a new 303
Mitchell's, Mr. case of gout 244
Moore, Mr. on eau medicinale 224
Mortality, bill of 128
Moth, superstition respecting the death's head 349
Mountains, description of remarkable 11
National institute, report of the 420
Naturalist's monthly reports 85, 173, 261, 349, 427, 515
Nervous affection, case of 459
New York, account of the botanical garden at 162
———, medical society at 421
Noli me tangere, case of 503
Nott's, Dr. posological companion, account of 246
Nottingham medical report 151
Nysten, Dr. on gases injected into the blood vessels 251
———, review of his physiological researches 405
Oleum terebinth in tape worm, on 31
Opthalmia, description of 152
Opium in rheumatism, on 210
Opossum, memoirs on the 422
Orange trees, introduction of 475
Os pubis, case of fractured 376
Oximuriatic gas and oxigen, combination of 298
Paralysis, case of apparent 153
Paris, Dr. on the physiology of the egg 7
Parkinson, Mr. on madhouses 148
Parry, Dr. on nervous affection 459
Parturition, rupture of the lungs after 166
Pearson, Dr. on quicksilver 29
———, Mr. on vaccination at Canton 453
Pericardium, aneurism of the aorta which bursts into the 251
Petty, Sir William, remark of 4
Pharmacopœia, of the London 21
Physic, history of primitive 5
Physicians, on the practices of 385, 455
———, on travelling 420
Physiologie, recherches de 405
Plants, on the organs of 8
———, effects of arsenic on 31
Poison of the upas, on the 252
Poisoning by foxglove, case of 155
Poisons, action of vegetable 129
Polypus vaginæ, case of 38
Popliteal aneurism, on 241
Portsmouth, new Hampshire bill of mortality at 127
Prepuce, on herpes of the 495
Ptyalism, case of spontaneous 452
Puerperal fever, on 265
Purging in the gout, on 244
Quacks, observations on 124
Quality inherent in inoculated small pox 117
Query on the eau medicinale 385
Questions, prize 76, 168, 169, 345
Quicksilver, remarkable circumstance concerning 29
Ramsey's, Mr. case of erythema mercuriale 341
Ramsbotham, Dr. on puerperal fever 265
Reade, Dr. on epiphora 227
Reform, on medical 2, 123
Reid, Dr. on the study of medicine 68
Report of the national vaccine establishment 234
——— Broad-street vaccine institution 488
Rheumatism, on calomel and opium in 210

### INDEX.

Rheumatism, effect of mercury in acute 277
——, observations on acute 449
Rhododendrum chrysanthum, its use in gout 408
Rhubarb in gout, on 499
Ring, Mr. on Cathartics in gout 380
——, review of his treatise on gout 408
Ringworm of the scalp, on the 42, 264
Royal society, proceedings of the 167, 247
Rubia tinctorum, virtues of 105
Rupture of the lungs in parturition 161
—— of the spleen and liver 337
Salt, on the varieties of 19
Sassafras, description of the nut of 244
Scalp on the ring-worm of the 42
Scarlatina, observations on 256, 348
—— anginosa, cases of 443
Sensation, theory of 89, 353, 432
——, observations on 484
Simpson's tooth instrument, described 167
Small pox, on the contagion of the inoculated 117, 207
——, cases of secondary 83, 236, 379, 423
——, after vaccination 82, 177, 234, 249
——, extirpated 35
——, deaths by 509
Smith's, Mr. theory of sensation 89, 353, 432
——, observations on 280
—— observations on lithotomy 285
Snake in the eye of a horse 273
Society for the relief of the widows of medical men 457, 510
Societies, proceedings of learned 76, 167, 168, 169, 247, 420, 421, 504
Sodalite, analysis of 303
Somnambulism, case of 483
Sound, experiments on 168
Spalding's, Dr. bill of mortality 127
Spine, case of fractured 371
Spleen, case of ruptured 337
Stone, on the operation for the 72
Storers's, Dr. case of small pox 378
Stramonium, observations on 22, 48, 150, 490
Sugar in diabetic blood, on 462
Surgery, present state of 33
Surgical anatomy, observations on 388
Swallows, utility of 174
Syncopo, case of 154
Syphilis, observations on 423
Tape worm, efficacy of ol. tereb. in 31
Tegart's, Mr. case of small pox 197
Tetanus in America, observations on 402, 428
Thigh, on the treatment of fractured 120
Thomson's, Dr. analysis of sodalite 303
——, Mr. London Dispensatory 327, 394
Thornton's, Dr. botanical lottery 345
Throat, case of burn in the 158
Tic douloureux, on 494
Tinea capitis, case of 203
Toads, singular notion concerning 427
Tobacco, experiments with 134, 137
Tongue, case of tumours on the 494
——, on poisons applied to the 129
Tooth instrument, the description of a new 167
Travers, Mr. on wounds in the cavity of the body 167
Trees, observations on 8
Trye, Mr. on operations for the stone 72
——, memoir of 407
Twin cases, observations on 115, 282
Typhus, observations on 37
Ulcer in the forehead, cases of 364, 5
Upas, on the poison of the 252
—— antiar, experiments with the 140
Urine, larvæ of an insect found in 155
——, case of suppression of 161
——, experiments on the 407
Vaccination, cases of small pox after 82, 177, 234, 249
——, state of 35
——, account of the progress of 389
——, reports on 234, 416, 488
—— at Canton 453
—— in New South Wales 518
Valentine, Dr. on tetanus 482
Vectis, on the 111
Vegetable poisons, observations on 129
—— physiology, on 170
Vena saphena major, operation for 508
Vessels in inflammation, on the action of 353
Vision, observations on 247
Vital power, on the term 441
Want, Mr. on gout 188
Water, observations on 394
Weever, remedy for the sting of the 259
Wells, Dr. on vision 247
Wernerian society, proceedings of the 168
Whately, Mr. on aqueous injections 110
Wigan, Dr. on croup 509
Wine, on the alcohol of 168
Woodham, Mr. on sensation 284
Woolaston, Dr. on diabetes 462
Woorara, experiments with the 139
Woolhouse, J. T. account of 220
Worms, on the theory of 273
Wounds in the cavity of the body, on the 167

### REFERENCES TO THE PLATES.

Case of Polypus Vaginæ - - - - - Page 38

Case of Fractured Spine - - - - - 371

Improved Tooth Instrument - - - - 167

